# Comparison of the i-gel and other supraglottic airways in adult manikin studies: Systematic review and meta-analysis

**DOI:** 10.1097/MD.0000000000005801

**Published:** 2017-01-10

**Authors:** Jiwon An, Sang Beom Nam, Jong Seok Lee, Jinae Lee, Hanna Yoo, Hye Mi Lee, Min-Soo Kim

**Affiliations:** a Department of Anesthesiology and Pain Medicine, Anesthesia and Pain Research Institute; b Biostatistics Collaboration Unit, Yonsei University College of Medicine, Seoul, Republic of Korea.

**Keywords:** adult, airway management, laryngeal masks, manikins

## Abstract

**Background:**

The i-gel has a gel-like cuff composed of thermoplastic elastomer that does not require cuff inflation. As the elimination of cuff inflation may shorten insertion time, the i-gel might be a useful tool in emergency situations requiring prompt airway care. This systematic review and meta-analysis of previous adult manikin studies for inexperienced personnel was performed to compare the i-gel with other supraglottic airways.

**Methods:**

We searched PubMed, the Cochrane Library, and EMBASE for eligible randomized controlled trials (RCTs) published before June 2015, including with a crossover design, using the following search terms: “i-gel,” “igel,” “simulation,” “manikin,” “manikins,” “mannequin,” and “mannequins.” The primary outcomes of this review were device insertion time and the first-attempt success rate of insertion.

**Results:**

A total of 14 RCTs were included. At the initial assessment without difficult circumstances, the i-gel had a significantly shorter insertion time than the LMA Classic, LMA Fastrach, LMA Proseal, LMA Unique, laryngeal tube, Combitube, and EasyTube. However, a faster insertion time of the i-gel was not observed in comparisons with the LMA Supreme, aura-i, and air-Q. In addition, the i-gel did not show the better results for the insertion success rate when compared to other devices.

**Conclusion:**

The findings of this meta-analysis indicated that inexperienced volunteers placed the i-gel more rapidly than other supraglottic airways with the exception of the LMA Supreme, aura-i, and air-Q in manikin studies. However, the quicker insertion time is clinically not relevant. The unapparent advantage regarding the insertion success rate and the inherent limitations of the simulation setting indicated that additional evidence is necessary to confirm these advantages of the i-gel in an emergency setting.

## 1 Introduction

In emergency situations such as sudden cardiac arrest and apnea, securing the airway for ventilation and oxygenation is a critical life-saving procedure.^[1]^ In this regard, endotracheal intubation (ETI; Mallinckrodt, Athlone, Ireland) has been deemed as the optimal method for ensuring a safe and patent airway. However, ETI requires highly skilled operators and may result in prolonged interruptions of resuscitation and serious complications including elevation of intracranial pressure and unrecognized esophageal intubation.^[2]^ During cardiopulmonary resuscitation (CPR), a pause of chest compression for placement of airway devices should be brief to preserve tissue perfusion.^[3]^ To minimize interruptions of chest compression and catastrophic events from intubation efforts, the use of a supraglottic airway has been considered as an alternative to ETI due to its technical ease and reduction of invasiveness.^[1,4,5]^

A variety of supraglottic airways have been introduced in the field of anesthesia and emergency situations. Compared to most supraglottic airways with an inflatable cuff, the i-gel (Intersurgical Ltd.; Workingham, UK) has a gel-like cuff composed of thermoplastic elastomer that does not require inflation.^[6,7]^ If successful insertion could be established in shorter insertion time by omitting cuff inflation, the i-gel might be a more valid device in emergency settings requiring prompt airway management. Several previous studies supported this hypothesis.^[8,9]^ However, meta-analyses in the field of anesthesia did not show consistent results with regard to the insertion time of the i-gel.^[6,7,10]^

Simulation with manikins has been performed widely for education and research related to airway management.^[11,12]^ Currently, there have been several comparative studies using manikins in order to identify the optimal supraglottic airway for various groups of healthcare providers under emergency situations.^[11,13,14]^ This systematic review and meta-analysis of previous adult manikin studies for inexperienced personnel was performed to compare the i-gel with other supraglottic airways.

## 2 Methods

Our systematic review and meta-analysis was performed according to the Preferred Reporting Items for Systematic Reviews and Meta-Analyses recommendations.^[15]^ The protocol for this study was registered with PROSPERO (registration number: CRD42015024290). Ethics committee is not applicable in this meta-analysis.

### 2.1 Data sources and search strategy

We included prospective randomized controlled trials (RCTs) published before June 2015, including those with a crossover design, that utilized adult-sized manikins to compare the i-gel with any other type of supraglottic airway. Two authors (JA and M-SK) independently performed database searches in PubMed, the Cochrane Library, and EMBASE for eligible simulation trials using the following search terms: “i-gel,” “igel,” “simulation,” “manikin,” “manikins,” “mannequin,” and “mannequins.” Language restrictions were not imposed in our searches. Studies involving anesthesiologists were excluded from our reviews. Disagreements over the inclusion or exclusion of studies were resolved by the final opinion of a third author (JSL). References cited in the included articles were also investigated to discover potentially eligible trials.

### 2.2 Data extraction

From the included trials, 2 authors (SBN and HML) independently extracted the following data: name of the first author, year of publication, journal name, study design, participant characteristics and number, presence of concurrent chest compression or difficult situations, and outcomes including insertion time and insertion success rate. The primary outcomes of this review were device insertion time and the first-attempt success rate of insertion at the initial assessment in difficult situations. Additional outcomes such as outcomes obtained at the second assessment or under difficult circumstances, the overall insertion success rate, and device preference were included as secondary outcomes. The first-attempt or overall success rate was determined in accordance with the definitions of insertion failure (e.g., time limitations and numbers of insertion attempts) described in each study. When the values were presented as median and total range, or an interquartile range of values, the mean value was estimated from the devised formula using the values of the median and the high and low ends of the range for less than 25 samples, and the median value itself was regarded as the mean value for more than 25 samples. The standard deviation was estimated from the devised formula using the values of the median and high and low ends of the range for less than 15 samples, the values of the range/4 for 15 to 70 samples, and the values of the range/6 for more than 70 samples. When only an interquartile range was provided from the selected articles, the standard deviation was calculated using the interquartile range/1.35.^[16,17]^

### 2.3 Risk of bias assessment

Two authors (JA and JSL) evaluated the risks of bias in the selected articles according to the Cochrane Collaboration's tool consisting of selection, performance, detection, attrition, reporting, and other sources of bias.^[18]^ The bias was graded as “low risk,” “high risk,” or “unclear.”

### 2.4 Statistical analysis

All analyses were conducted with Comprehensive Meta-Analysis software (version 2.0, Biostat; Englewood, NJ) and R statistical software (version 3.2.3; R Foundation for Statistical Computing, Vienna, Austria, https://www.r-project.org), and all statistical results are presented with 95% confidence intervals (CIs). In continuous variables such as insertion time, we calculated mean difference (MD) at the individual study level and the pooled MD using the inverse variance method in a fixed-effect model or the DerSimonian–Laird (D–L) method in a random-effects model. In dichotomous variables, we calculated the relative risk (RR) at the individual study level and the pooled RR using the Mantel–Haenszel (M–H) method in a fixed-effect model or the D–L method in a random-effects model. After discussion with a medical statistician, the correlation coefficient between devices for meta-analysis of crossover comparisons was estimated as 0.5.^[19]^ In addition, we chose the smaller value when the sample sizes of 2 device groups under crossover trials were different. The *Q* test and chi-squared test were performed to assess heterogeneity. Substantial heterogeneity of the effect sizes was defined as an *I*^2^ value of more than 50% or a *P* value of <0.10 on the chi-squared test, for which we applied a random-effect model instead of a fixed-effect model. When applying the random-effect model in data sets containing 3 or more individual studies, the goodness-of-fit test based on the Shapiro–Wilk test was performed to check the adequacy of the random-effect model. A *P* value of <0.05 suggested that the use of a random-effect model would not be appropriate.^[20]^

Visual assessments of funnel plots and Egger linear regression tests were conducted to confirm the possibility of publication bias. Asymmetry in funnel plots and a *P* value of <0.10 on Egger test suggested the presence of publication bias.

## 3 Results

### 3.1 Eligible studies and study characteristics

**Figure 1:**
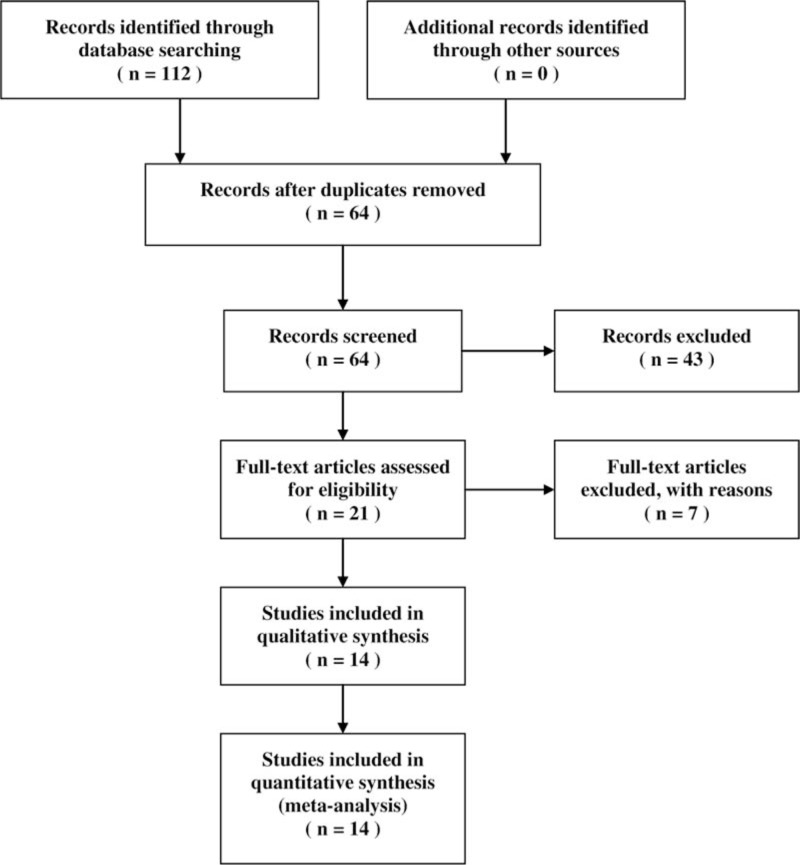
Flow diagram showing data searches and article selection.

We performed electronic database searches and included 14 full-text articles, as shown in Fig. 1.^[1,4,11,13,14,21–29]^ All studies were randomized crossover trials except 1 with parallel design.^[21]^ The included articles contained several comparisons between the i-gel and other supraglottic airways as follows: 4 comparisons with the LMA Classic (LMA North America, Inc., San Diego, USA),^[11,22,25,29]^ 5 with the LMA Fastrach (Laryngeal Mask, Prodol Meditec, Spain),^[13,14,24,27,28]^ 3 with the LMA Proseal (LMA North America, Inc., San Diego, USA),^[1,14,23]^ 3 with the LMA Supreme,^[13,23,24]^ 5 with the LMA Unique (LMA North America, Inc., San Diego, USA),^[1,4,14,23,24]^ 7 with the laryngeal tube (King-LT-D, VBM, Sulz, Germany),^[1,4,13,14,21,22,24]^ 5 with the Combitube (Covidien, Mansfield, MA, USA),^[1,4,13,14,24]^ 4 with the EasyTube (Teleflexmedical Ruesch, Research Triangle Park, NC, USA),^[1,4,13,24]^ 1 with the SoftSeal (Smiths Medical International Ltd, Ashford, Kent, UK),^[26]^ 1 with the AuraOnce (Ambu, Ballerup, Denmark),^[26]^ 1 with the aura-i,^[27]^ and 1 with the air-Q (Cookgas LLC, Mercury Medical, USA).^[27]^ Characteristics of the included articles are summarized in Table 1 . In 4 studies, the evaluation was repeated after 3 or 12 months.^[1,13,23,24]^ Four studies included insertions of devices under difficult circumstances, such as while wearing protective equipment and applying a neck collar and pathologic airway conditions.^[14,24,25,29]^ In this meta-analysis, insertion variables investigated at the second assessment or under difficult circumstances were analyzed additionally. Robak et al's^[24]^ study assessed device insertion under simulated physiologic and pathologic airway conditions at the initial and second assessments. However, the insertion success rate under physiologic conditions was only included in this analysis as its exact values investigated under pathologic airway conditions were not stated at both of the assessments. In addition, the insertion times measured under pathologic airway conditions at each assessment time were regarded and analyzed as those from a single study. Ongoing chest compression during device insertion was applied in 4 studies.^[4,26,27,29]^ However, Ruetzler et al's^[4]^ study allowed transient interruption of chest compression during airway management as required by participants. In Adelborg et al's^[26]^ study, we could not confirm outcomes during concurrent chest compression. Komasawa et al's^[27]^ study assessed the insertion of devices under both situations with and without chest compression. When conducting analyses using this study, the outcomes obtained from each situation were regarded and analyzed as those from a single study.

**Table 1:**
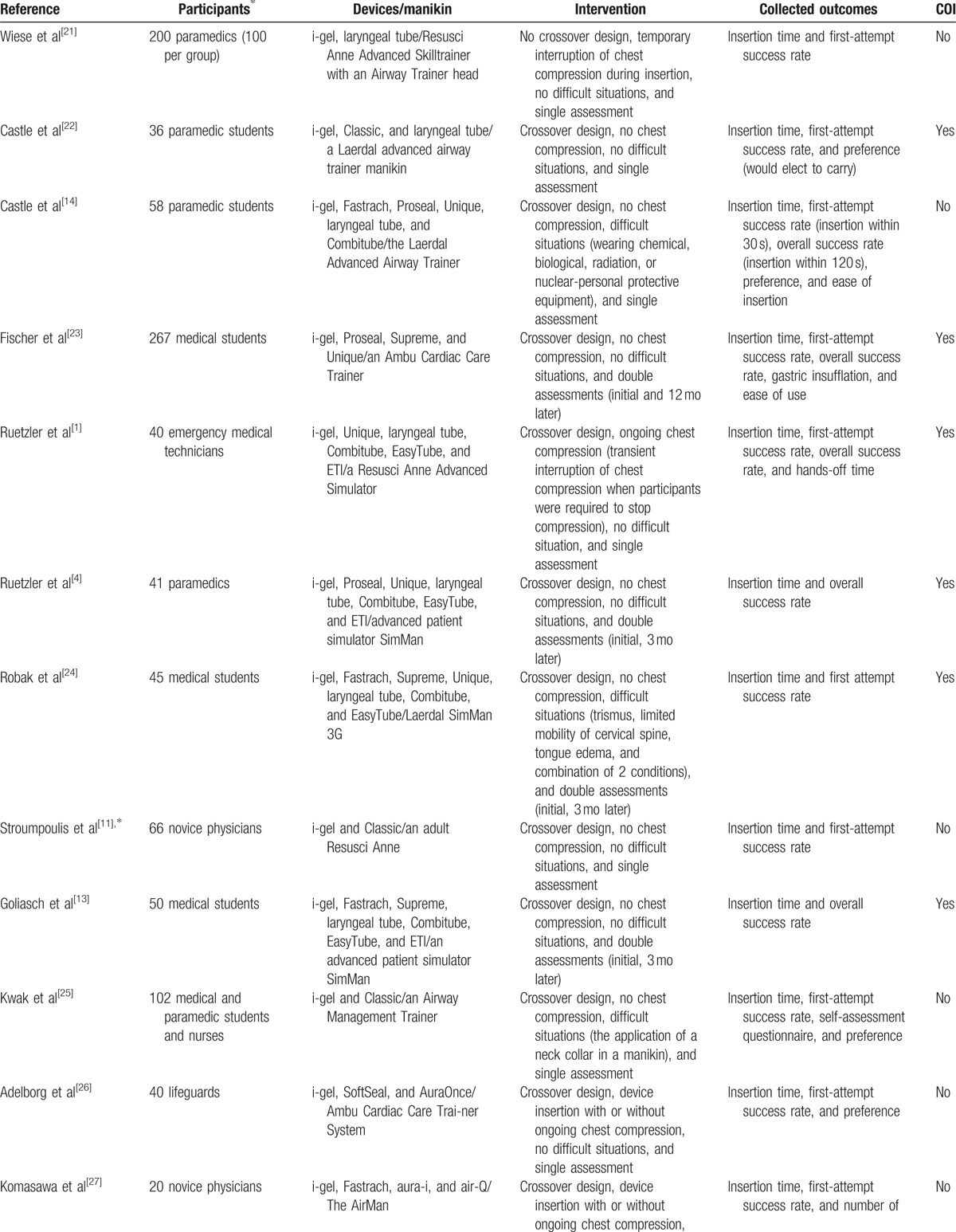
Characteristics of the included RCTs.

**Table 1 (Continued):**
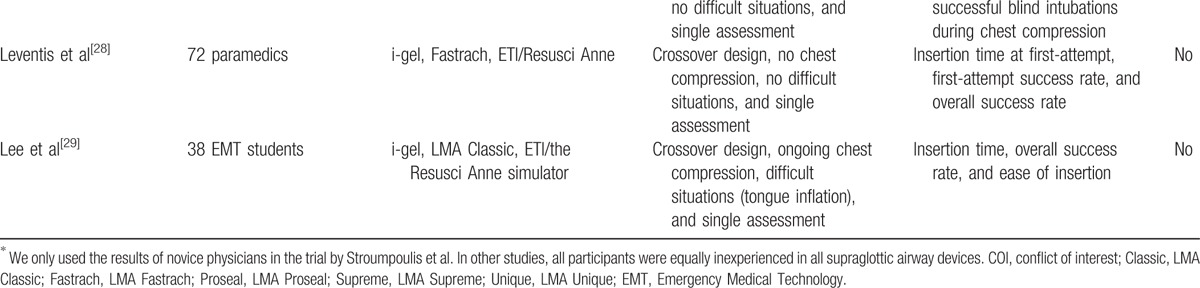
Characteristics of the included RCTs.

Considering the aim of this meta-analysis and the level of participants’ experience in other trials, we only used the results of novice physicians in the trial by Stroumpoulis et al.^[11]^ Castle et al's^[14]^ 2011 study provided insertion success rates according to certain time periods, and a successful insertion rate within 30 seconds was regarded as the first-attempt success rate. The insertion success rate from Komasawa et al's^[27]^ study was also regarded as a first-attempt success rate as the success or failure of insertion within 30 seconds was investigated. Leventis et al's^[28]^ study presented insertion times and success rates at each attempt of device placement, and we selected the results at the first-attempt insertion for this analysis.

### 3.2 Risk of bias assessment

Risks of bias are presented in Table 2. In all enrolled studies, performance and detection bias regarding the blinding of participants and assessors was graded as high risk. Conflicts of interest were reported in 6 trials,^[1,4,13,22–24]^ and regarded as other bias.^[30]^

**Table 2:**
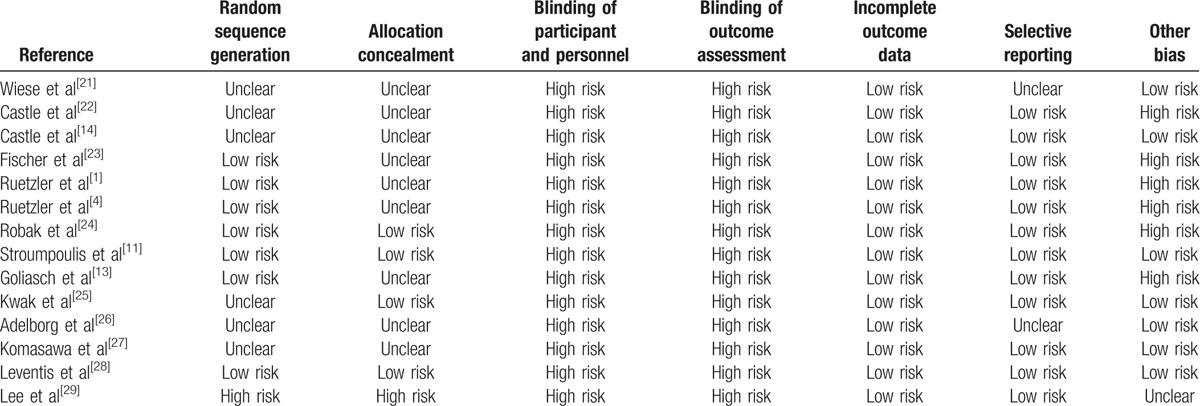
Risk of bias assessment.

### 3.3 Meta-analysis of primary outcome measures

The insertion times at the initial assessment without difficult circumstances were obtained from all of the included studies. The forest plots regarding regarding pooled analyses of the i-gel and each other supraglottic airway with 2 or more comparisons from the included studies were provided in Fig. 2 . The i-gel had a significantly shorter insertion time than the LMA Classic, LMA Fastrach, LMA Proseal, LMA Unique, laryngeal tube, Combitube, and EasyTube. However, a faster insertion time of the i-gel was not observed in comparisons with the LMA Supreme, aura-i, and air-Q. In SoftSeal and AuraOnce with only 1 comparison, the insertion time of the i-gel was significantly faster than both of the devices (MD −19.60, 95% CI −21.53 to −17.67; MD −19.50, 95% CI −21.80 to −17.20, respectively). Overall analysis of the i-gel and all other supraglottic devices showed that the i-gel reduced mean (95% CI) insertion time by −8.09 (−9.70, −6.47) seconds compared to other supraglottic airways (*P* < 0.001), but there was substantial heterogeneity (*I*^2^ = 99%).

**Figure 2:**
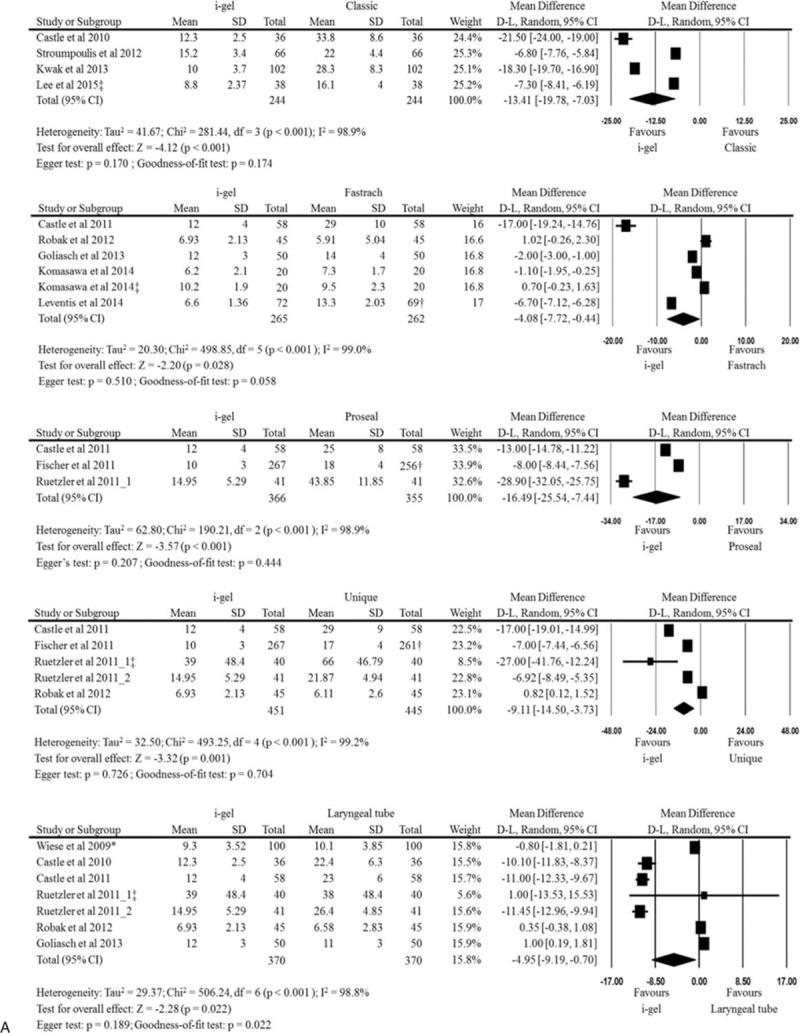
Forest plots denoting comparisons of insertion time at the initial assessment without difficult circumstances between the i-gel and other supraglottic airways. D–L = DerSimonian–Laird. ^†^analysis with smaller sample size; ^‡^insertion during chest compression.

**Figure 2 (Continued):**
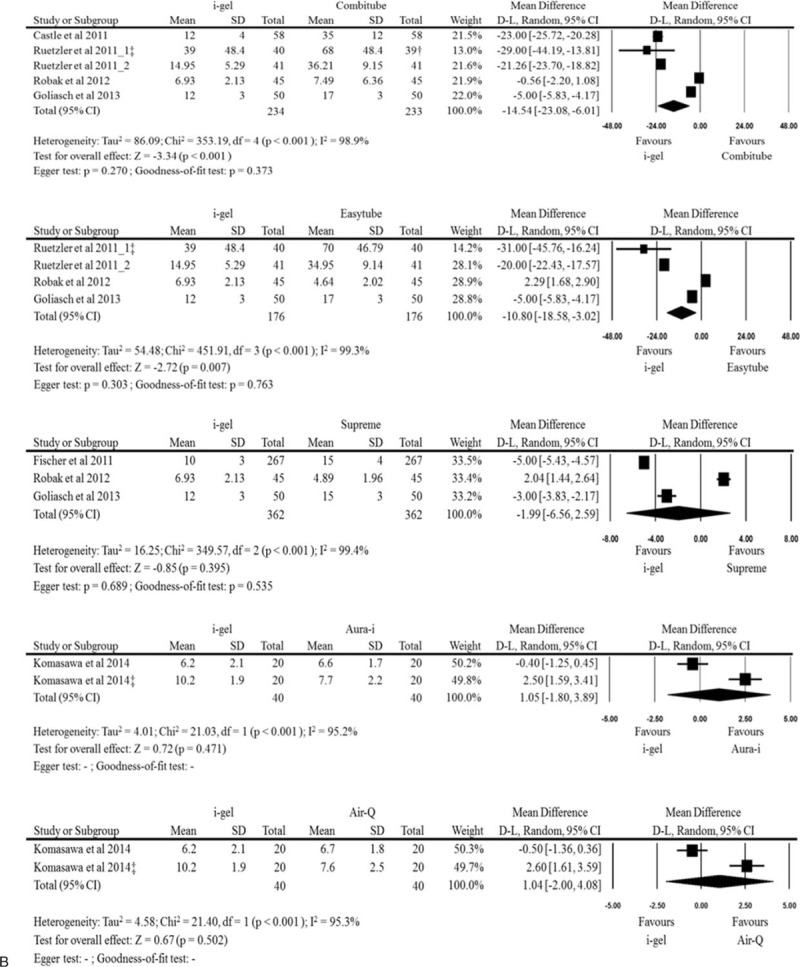
Forest plots denoting comparisons of insertion time at the initial assessment without difficult circumstances between the i-gel and other supraglottic airways. D–L = DerSimonian–Laird. ^†^analysis with smaller sample size; ^‡^insertion during chest compression.

Data regarding the success rates of the first-attempt insertion at the initial assessment without difficult circumstances were obtained from 11 studies.^[4,11,14,21–28]^ In the 2 included studies,^[22,24]^ all of the device insertions were successfully established at the first attempt. The meta-analysis results of the first-attempt insertion success rate from the remaining 9 studies are described in Table 3. The i-gel had a significantly lower success rate than the LMA Supreme and EasyTube.

**Table 3:**
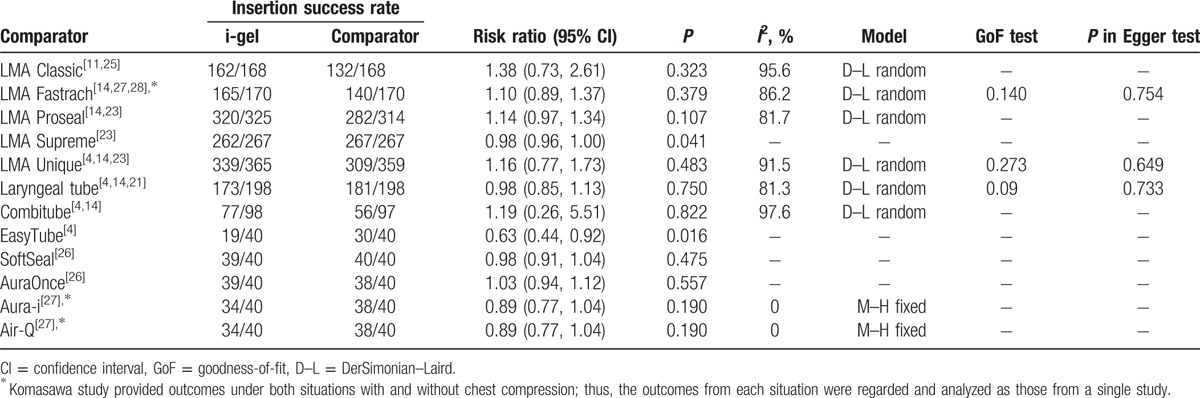
Meta-analysis of the first-attempt insertion success rate at the initial assessment without difficult circumstances between the i-gel and other supraglottic airways.

### 3.4 Meta-analysis of secondary outcome measures

Data regarding insertion times obtained at the second assessment without difficult circumstances were collected from 4 studies.^[1,13,23,24]^ Significantly faster insertion of the i-gel was confirmed only in comparisons with the laryngeal tube and Combitube (Table 4).

**Table 4:**
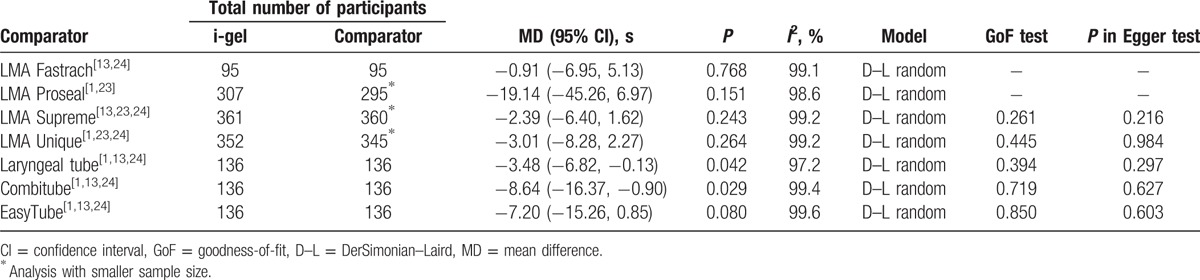
Meta-analysis of insertion time at the second assessment without difficult circumstances between the i-gel and other supraglottic airways.

Four studies reported the insertion times obtained under difficult circumstances at the initial or second assessment.^[14,24,25,29]^ Meta-analysis outcomes are demonstrated in Table 5. The i-gel was placed more quickly than the LMA Classic and LMA Proseal, whereas significantly slower insertion of the i-gel was observed in comparisons with the EasyTube and LMA Supreme.

**Table 5:**
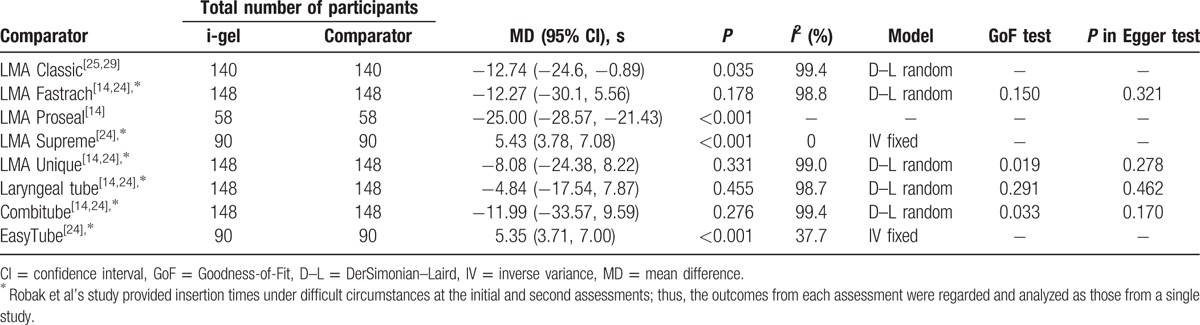
Meta-analysis of insertion time under difficult circumstances between the i-gel and other supraglottic airways.

The first-attempt insertion success rates at the second assessment without difficult circumstances were provided in 2 of the included studies.^[23,24]^ In 1 study,^[24]^ all devices were placed successfully at the first attempt. In the other study,^[23]^ the success rate of the i-gel was the higher than those of the LMA Proseal and LMA Unique (RR 1.32, 95% CI 1.20–1.45; RR 1.33, 95% CI 1.21–1.47, respectively), whereas it was the lower than that of the LMA Supreme (RR 0.94, 95% CI 0.89–1.00).

The first-attempt insertion success rates under difficult circumstances were reported in 2 of the included studies.^[14,25]^ One study reported a superior success rate of the i-gel when compared with those of the LMA Fastrach, LMA Proseal, LMA Unique, laryngeal tube, and Combitube.^[14]^ In the other study, there was no significant difference in the success rate between the i-gel and LMA Classic (RR 1.02, 95% CI 0.99–1.05).^[25]^

Overall insertion success rates were collected from 7 studies.^[1,4,13,14,23,28,29]^ There were no significant differences in the overall success rate between the i-gel and other devices, except the LMA Proseal^[1,23]^ and LMA Unique^[23]^ at the second assessment without difficult circumstances (RR 1.13, 95% CI 1.00–1.28; RR 1.11, 95% CI 1.06–1.16, respectively).

### 3.5 Ancillary results from systemic review

Participants were asked about device preference in 4 studies,^[14,22,25,26]^ and the i-gel was the most preferred device in all surveys. Ease or difficulty of device use was investigated in 3 studies.^[14,23,29]^ In Fisher study, the i-gel and LMA Supreme together were graded as easier to use compared to the LMA Unique and LMA Proseal.^[23]^ In the remaining 2 studies, the i-gel received better scores than other devices.^[14,29]^ One study evaluated gastric insufflation after insertion,^[23]^ and the i-gel had the lowest rate (0%) at initial testing and a significantly increased rate (4%) at second testing after 12 months. From this study, the LMA Supreme showed the best results regarding gastric insufflation (1% at initial testing and 2% at second testing). Success rates of blind intubation with 4 supraglottic airways under ongoing chest compression were evaluated in 1 study.^[27]^ The highest success rate was observed in the air-Q (15 of 19) compared to the aura-i (14 of 19), i-gel (12 of 16), and LMA Fastrach (10 of 18).

## 4 Discussion

Our systematic review and meta-analysis revealed that the insertion time of the i-gel was significantly shorter than those of the LMA Classic, LMA Fastrach, LMA Proseal, LMA Unique, laryngeal tube, Combitube, and EasyTube in the initial assessment without difficult circumstances. However, the superiority of the first attempt and overall success rates of insertion in the i-gel was not apparent.

Speed of insertion is the most important prerequisite when selecting a supraglottic airway for securing airway patency during CPR, as recent resuscitation guidelines emphasize that chest compressions should be interrupted briefly for placement of airway devices.^[3,4,31]^ The i-gel can be considered as a reasonable candidate for meeting this requirement due to its noninflatable cuff and design for easy insertion.^[32]^ Meta-analyses to secure evidence for the superiority of the i-gel have been carried out primarily in the field of anesthesia. A meta-analysis of the adult-sized i-gel under general anesthesia demonstrated that the i-gel had a shorter insertion time than other devices.^[7]^ However, heterogeneity in the pooled results of insertion time was substantial, and faster insertion times of the i-gel were not observed in subgroup analyses of second-generation devices including the LMA Proseal and LMA Supreme. In a meta-analysis of pediatric patients under general anesthesia, a pooled analysis did not show a significant difference in insertion time between the i-gel and different types of LMA devices, and the i-gel had an inferior result in a subgroup analysis with the LMA Supreme and AuraOnce (MD 1.69 seconds, 95% CI 0.25–3.13, *P* = 0.02, *I*^2^ = 27%).^[6]^ Chen et al^[10]^ performed a meta-analysis using studies comparing the i-gel and LMA Supreme, and no differences in device placement time were found in a pooled analysis of the 2 devices.

From our systemic review, the i-gel was inserted more rapidly than other devices in most of the included papers. However, several studies reported contradictory results that may have been responsible for the heterogeneity of the meta-analyses outcomes.^[24,27]^ Robak et al^[24]^ reported that the insertion time of the i-gel was greater in most of the comparisons, while in Komasawa et al's^[27]^ study, the time required for i-gel insertion without chest compression was shorter than those of the LMA Fastrach, aura-i, and air-Q. When applying chest compression, the i-gel insertion time was lengthened significantly, resulting in the i-gel showing the longest insertion time under chest compression. The authors commented that these results might have been due to the anatomically curved shaft that is present on the other 3 supraglottic airways yet absent from the i-gel.^[27]^ Under anesthesia, the LMA Supreme also showed similar insertion times when compared to the i-gel.^[6,7,10]^ From our meta-analyses, a faster insertion time of the i-gel was not observed in comparisons with the LMA Supreme. The LMA Supreme also has a semirigid and anatomically curved airway tube.^[10]^ Thus, the morphologically improved airway tube of supraglottic airways, such as a noninflatable cuff, may also reduce the insertion time.

The success rate of first-attempt insertions is also a significant selection criterion for choosing the supraglottic airway in an emergency setting.^[33]^ From the abovementioned meta-analyses in the anesthesia area, the insertion success rate at the first attempt for the i-gel was similar to those of other devices.^[6,7,10]^ Maitra et al^[17]^ also evaluated the i-gel in children via meta-analysis, and no difference was observed in the first-insertion success rate. In the present meta-analysis, the superiority of the insertion success rate at the first attempt for the i-gel was not apparent. In addition, the i-gel had a lower success rate when compared with the LMA Supreme (RR 0.98, 95% CI 0.96–1.00, *P* = 0.041), although there was only 1 comparison between the 2 devices.^[23]^ Ragazzi et al^[34]^ reported a better first-attempt success rate, a higher sealing pressure, and fewer failures with the LMA Supreme than with the i-gel when devices were inserted into patients under general anesthesia by inexperienced operators. Hence, additional studies regarding the feasibility of supraglottic airways with an anatomically curved shaft such as the LMA Supreme are needed in order to select the most adequate airway in an emergency setting.

Our meta-analysis had several limitations that must be considered when interpreting the results. First, there was considerable heterogeneity in most of the meta-analyses. Essentially, the heterogeneity could have originated from methodological differences among the included studies (which had parallel or crossover designs), the definition of insertion time, and the presence of chest compression or difficult situations. In particular, the use of several types of manikins could also affect the insertion performance of each supraglottic airway.^[35]^ In addition, the study participants of various occupations have different experiences each other for supraglottic airway. These differences in degree of education or experience related to supraglottic airways could have been the most likely cause of these heterogeneous results, given that various groups including paramedics, students, nurses, and physicians inserted the devices in each study. The more variable MDs of insertion times in the simulation studies included in this meta-analysis (–31 to 5.4 seconds) might support this assumption, particularly when compared to the studies on insertion under general anesthesia (–11 to 3 seconds).^[6,7,10]^ Second, information on sealing function from the included simulation trials was not present except in 1 trial assessing gastric insufflation.^[23]^ Adequate sealing function in supraglottic airways, which is mainly evaluated by measuring the oropharyngeal leak pressure is important for maintaining ventilation and protecting the airway from secretions.^[17]^ Given that the oropharyngeal leak pressure of the i-gel assessed under anesthesia was similar or superior to those of other devices,^[7,17]^ it was expected that the sealing function of the i-gel was also acceptable under emergency situations. Third, an airway model using a manikin is not a clinical model; thus, results from manikin studies may not be valid. However, simulation with manikins allows for stricter control of possible confounding factors when conducting a study.^[11]^ In addition, a prospective study that compares and validates airway devices in an emergency setting may not be easy to perform using human subjects. Fourth, none of the included studies were free from performance or detection bias. Blinding of participants and assessors was impossible due to the methodology used in simulation studies with supraglottic airways.^[17]^ Lastly, as we were unable to calculate correlation coefficients for our enrolled crossover studies, a fixed cutoff value was used for our meta-analysis.

In conclusion, current evidence based on simulation trials implied that inexperienced volunteers placed the i-gel into the manikins more rapidly than other supraglottic airways, except the LMA Supreme, aura-i, and air-Q. Within the limitations of the simulation setting using manikins, these results suggest that the i-gel is more beneficial for inexperienced medical personnel under sudden emergency situations. However, the quicker insertion time is clinically not relevant. The unapparent advantage regarding the insertion success rate and the substantial heterogeneity in our meta-analysis indicated that additional evidence is necessary to confirm these advantages of the i-gel in an emergency setting.
